# Anatomical predispositions for silent cerebral infarction postcarotid artery stenting: a retrospective cohort

**DOI:** 10.1097/JS9.0000000000001833

**Published:** 2024-06-19

**Authors:** Tianhua Li, Renjie Yang, Jie Wang, Tao Wang, Guangjie Liu, Jiaqi Jin, Xuesong Bai, Ran Xu, Taoyuan Lu, Yabing Wang, Adam A. Dmytriw, Bin Yang, Liqun Jiao

**Affiliations:** a Department of Neurosurgery, Xuanwu Hospital, Capital Medical University, Xicheng District; b China International Neuroscience Institute (China-INI); c Department of Interventional Neuroradiology, Xuanwu Hospital, Capital Medical University, Beijing; d Department of Neurosurgery, Nanfang Hospital, Southern Medical University, Guangzhou, People’s Republic of China; e Department of Medical Imaging, University of Toronto, Diagnostic and Interventional Neuroradiology, Toronto, Ontario, Canada; f Harvard Medical School, Neuroradiology & Neurointervention Service, Brigham and Women's Hospital, Boston, Massachusetts, USA

**Keywords:** anatomical characteristics, carotid artery stenting, nomogram, silent cerebral infarction

## Abstract

**Background:**

Silent cerebral infarction (SCI) that manifests following carotid artery stenting (CAS) has been postulated to correlate with cognitive decline, the onset of dementia, and an increased risk of subsequent cerebrovascular events. This investigation aimed to thoroughly examine the potential anatomical predispositions that are linked to the occurrence of SCI post-CAS, and further develop a predictive nomogram that could accurately forecast the risk of SCI post-CAS.

**Methods:**

The present investigation conducted a retrospective examination of datasets from 250 individuals presenting with carotid artery stenosis who had been subjected to CAS within a tertiary healthcare institution from June 2020 to November 2021. Stratified by the procedural date, participants were allocated into a training cohort and a validation cohort. A nomogram was constructed predicated on salient prognostic determinants discerned via a multivariate logistic regression analysis.

**Results:**

An aggregate of 184 patients were incorporated into the study, of which 60 (32.6%) manifested SCI, whereas 124 (67.4%) did not. Within the training cohort (*n*=123), age (OR 1.08, 95% CI: 1.01–1.16; *P*=0.034), aortic arch type (Type III vs. I: OR 10.79, 95% CI: 2.12–54.81; *P*=0.005), aortic arch variant (OR 47.71, 95% CI: 6.05–376.09; *P*<0.001), common carotid artery (CCA) ostium lesions (OR 6.93, 95% CI: 1.49–32.32; *P*=0.014), and proximal tortuosity index (TI) (OR 1.01, 95% CI: 1.00–1.02; *P*=0.029) were demarcated as standalone risk predispositions for SCI subsequent to CAS. The concordance index (C-index) for the training cohort's nomogram stood at 0.89 (95% CI: 0.84–0.95). Moreover, the said nomogram exhibited commendable efficacy within the validation cohort (C-index=0.94) as well as the entire participant base (C-index=0.90). Furthermore, the decision curve analysis illustrated the exemplary clinical applicability of the nomogram.

**Conclusions:**

The findings of this inquiry underscore that age, aortic arch type, aortic arch variant, CCA ostium lesions, and proximal TI serve as independent determinants linked with SCI post-CAS. The formulated nomogram, predicated on these risk factors, possesses robust prognostic significance, and might serve as a valuable adjunct to inform clinical decision-making.

## Introduction

Highlights

- We found that age, aortic arch type, aortic arch variants, common carotid artery ostium lesions, and proximal tortuosity index were independent predispositions for silent cerebral infarction post carotid artery stenting.
- An accurate and efficient nomogram to predict silent cerebral infarction post carotid artery stenting was developed.
- The nomogram possesses robust prognostic significance and might serve as a valuable adjunct to inform clinical decision-making.

Carotid artery stenosis is responsible for 15–20% of all ischemic cerebrovascular events, imposing substantial morbidity upon affected individuals, their caregivers, and the broader societal milieu^1^. Coinciding with the maturation of interventional apparatuses and the proliferation of minimally invasive techniques, carotid artery stenting (CAS) has progressively been acknowledged as a viable surrogate to carotid endarterectomy in managing carotid stenosis^2^. Yet, CAS is juxtaposed with a pervasive incidence of silent cerebral infarction (SCI), a recent delineation indicates a prevalence spanning from 18 to 57%^3–7^. SCI has been found to have potentially serious and long-term clinical implications, including cognitive decline and dementia, as evidenced in previous studies^8,9^. Furthermore, the correlation between SCI and the risk of future cerebrovascular events remains a subject of significant concern and debate in the medical community^10–12^. Given the gravity of these issues, there has been a growing interest in elucidating the predisposing factors that contribute to SCI post-CAS.

Currently, the key to CAS is ‘how to reduce minor ischemic stroke and SCI’. Anatomical determinants serve as pivotal benchmarks in steering clinical undertakings in CAS^13^. Therefore, it is important to explore the relationship between anatomic characteristics and SCI to improve the prognosis of patients with carotid stenosis. Antecedent research endeavors have posited that a type III aortic arch and an elevated proximal tortuosity index (TI) might amplify the occurrence of SCI post-CAS, but the mechanism has not been elucidated^14–16^. In addition, the nexus between certain cardinal anatomical determinants (aortic arch variants, parameters of CCA, etc.) and SCI post-CAS remains uncharted. Prior investigations have accentuated the impact of discrete anatomical factors on SCI post-CAS, but the aggregated influence of these individual anatomical attributes remains elusive. In tandem, bespoke prognostic instruments anchored on vascular anatomical determinants to ascertain the likelihood of SCI post-CAS are conspicuously absent. Hence, it becomes imperative to discern and amalgamate potential anatomical determinant risk factors for SCI post-CAS and embark on tailored evaluations to edify CAS practitioners.

The objective of this inquiry was to decipher the potential predisposing factors of anatomical attributes for SCI post-CAS. Moreover, the endeavor aims to formulate a pioneering nomogram to furnish individualized therapeutic paradigms and stratagems for patients afflicted with carotid artery stenosis.

## Methods

The study received endorsement from the Ethics Committee of Xuanwu Hospital, Capital Medical University and comprehensive informed written assent was secured. Protocol identifier: [2021]124. Date of sanction: 2021-08-25. Registration unique identifying number: ChiCTR2100051697. The endeavor has been delineated in accordance with the strengthening the reporting of cohort, cross-sectional, and case–control studies in surgery (STROCSS) parameters^17^ (Supplemental Digital Content 1, http://links.lww.com/JS9/C803).

### Study population

Individuals presenting with carotid artery stenosis who underwent CAS through transfemoral access route at a tertiary healthcare institution in a tertiary healthcare institution in China were incorporated into the analysis spanning June 2020 to November 2021. Inclusion parameters were as follows: (1) individuals manifesting symptomatic carotid artery stenosis exhibiting angiographic stenosis surpassing 50% or individuals with asymptomatic carotid stenosis exhibiting angiographic stenosis exceeding 70%^18^; (2) complete preoperative computed tomography angiography (CTA) data. Exclusion parameters encompassed: (1) occluded internal carotid artery (ICA) or common carotid artery (CCA); (2) simultaneous revascularization of other lesion location; (3) the implicated vessel having undergone prior revascularization interventions; (4) individuals manifesting stroke, myocardial infarction, or untoward events culminating in mortality during the perioperative juncture.

### CAS protocol

CAS was instituted to mitigate cerebrovascular event risk in individuals presenting with moderate to severe ipsilateral carotid stenosis, as elucidated via catheter-assisted imaging or noninvasive diagnostic modalities. The concomitant risk determinants were managed in harmony with the American Heart Association/American Stroke Association directives^19^. Four types of stents were used in the study population, Wallstent (Boston Scientific), Precise (Johnson & Johnson), Acculink (Abbott Laboratories), and Protégé (Medtronic). Patients were counseled to administer a dual-antiplatelet regimen (comprising clopidogrel, 75 mg/day and aspirin, 100 mg/day) for a minimum span of 5 days antecedent to the intervention. Subsequent to the CAS, they were recommended to perpetuate the dual-antiplatelet regimen for 90 days, barring contraindications, and thereafter transition to a singular antiplatelet regimen indefinitely, predominantly aspirin (100 mg/day). During the intervention, subsequent to successful puncture and sheath positioning, heparin was administered calibrated at a dosage of body weight × 2/3 mg, succeeded an hour later by 1/2 of the initial dosage, and subsequently 1/2 of the antecedent dosage each succeeding hour. Preintervention or postintervention, all participants abstained from anticoagulation therapy barring the presence of alternate anticoagulation imperatives, such as atrial fibrillation or distal extremity venous thrombosis. All patients were prescribed postoperative statin therapy.

### Definition of SCI

MRI was executed 2 days prestenting and 2 days poststenting to evaluate emergent SCI. SCI was characterized by (1) the emergence of elevated signal during the cranial MRI DWI phase upon reevaluation 2 days postintervention, contrasted with antecedent operative documentation; (2) the absence of self-acknowledged neurological manifestations postoperation; (3) the lack of neurological localization indicators during postoperative transsystemic evaluation.

### Data collection

Baseline demographics for each participant were amassed, encompassing variables such as age, sex, whether the lesion is symptomatic, BMI, hypertension, diabetes, hyperlipidemia, coronary artery disease, atrial fibrillation, bronchial asthma, tobacco consumption, and alcohol intake. Their CTA was executed with slice intervals varying between 0.8 and 2 mm, ensuring optimal high-resolution imaging. Subsequently, three-dimensional reconstructions were formulated using the RadiAnt DICOM Viewer software suite (Medixant, Poland, version 5.5), facilitating meticulous visualization of vascular anatomy and pathophysiology. The intricate descriptors of the aortic arch included its typology, variants, and calcification status, as well as potential aberrations at the CCA ostium. Calcifications were stratified as mild, moderate, and pronounced based on <1/3, <2/3, and <1 proportional calcium accretions on the arterial facade^20^. Any deviations in the triad of principal branches of the aortic arch, namely the unnamed artery, the CCA, and the subclavian artery, were regarded as aortic arch variants^21,22^. The vertical demarcation between the apex of the arch and the genesis of the intended catheterized vessel—<1, 1–2, >2 cm—corresponded to type I, II, III aortic arch delineations, respectively^23,24^. The ensuing anatomical characteristics were scrutinized: the profundity and diameter of the CCA superior to the clavicle, the span of the CCA from the clavicle to the carotid bifurcation, the diameter of the internal ICA at the lesion focus and the cranial base, both proximal and distal tortuosity indices (TI), and the presence of tandem stenosis involving the CCA or middle cerebral artery (MCA). The TI was ascertained by aggregating angles deviating from the quintessential linear arterial trajectory. Proximal TI was gauged spanning from the aortic arch to the ICA stenosis, whereas distal TI was determined from the ICA stenosis to the intended deployment locus for the protective filter. The inception of the innominate artery or the left CCA from the arch was uniformly deemed 90°, irrespective of arch typology. A dyad of radiologists, boasting substantial expertise and domain-specific education, individually appraised each radiographic capture. In scenarios presenting evaluative discord, an elder radiologist rendered arbitration, facilitating a consensus-driven resolution.

### Sample size estimation

Power estimation methodology: normal approximation; alternative hypothesis: Two-sided; examination modality: Z-Test (unpooled); power: 0.8; significance level: 0.05; group apportionment: R=N2/N1=2. The incidence of SCI subsequent to CAS oscillated between 18 and 57%, averaging at 37.5%. P1 was established at 0.375, whereas P2 was designated 0.625. Upon computation, it was deduced that N1=45 and N2=90. Conclusively, this investigation encompassed 60 patients manifesting SCI and 124 non-SCI patients.

### Construction and validation of a nomogram

All 184 patients were categorized based on the operation date, with the initial 2/3 constituting the training cohort and the residual 1/3 comprising the validation cohort. Subsequently, the training cohort was bifurcated into SCI and normal groups. Within the training cohort, both univariate and multivariate logistic regressions were employed to scrutinize variables demonstrating statistically salient disparities and to ascertain the autonomous prognostic risk determinants for SCI post-CAS. Following this, a nomogram was synthesized predicated upon these risk determinants, and receiver operating characteristic (ROC) curves were delineated. The concordance index (C-index) in tandem with calibration curves served to evaluate the nomogram's discriminative capacity and calibration. The clinical relevance was ascertained via decision curve analysis (DCA) curves^25^. The efficacy of this model, in terms of construction, discrimination, and calibration, was subsequently assessed within both the validation and whole cohorts, utilizing the methodologies elucidated prior.

### Statistical analysis

Analytical procedures were executed utilizing SPSS 26.0 (IBM). Chi-square evaluations were employed to assess descriptive metrics, denoting enumerations (proportions) of categorical determinants. Continuous determinants underwent normality examination via the nonparametric one-sample Kolmogorov–Smirnov test. For those adhering to a normal distribution, the independent samples *t*-test was harnessed, with results articulated as mean±SD. Non-normally distributed continuous variables were articulated as median (25th–75th percentile) and scrutinized using the Mann–Whitney *U*-test. A statistical threshold was demarcated by a two-sided *P*-value <0.05. Comparison of baseline clinical variables in patients with and without SCI was compared using univariate logistic regression. Independent variables were included in the multivariate logistic regression model based on a *P*<0.05 on univariate analysis. Ensuing analyses were conducted utilizing R (4.2.1) software. The multivariate logistic regression framework was formulated via the glm function, the nomogram model was crafted using the rms (6.4.0) package, ROC examination was conducted employing the pROC (1.18.0) package, calibration scrutiny was facilitated using the rms (6.4.0) package, and the pertinent net return scenario was computed via the rmda (1.6) package. Results were visualized through ggplot2 (3.3.6).

## Results

**Figure 1:**
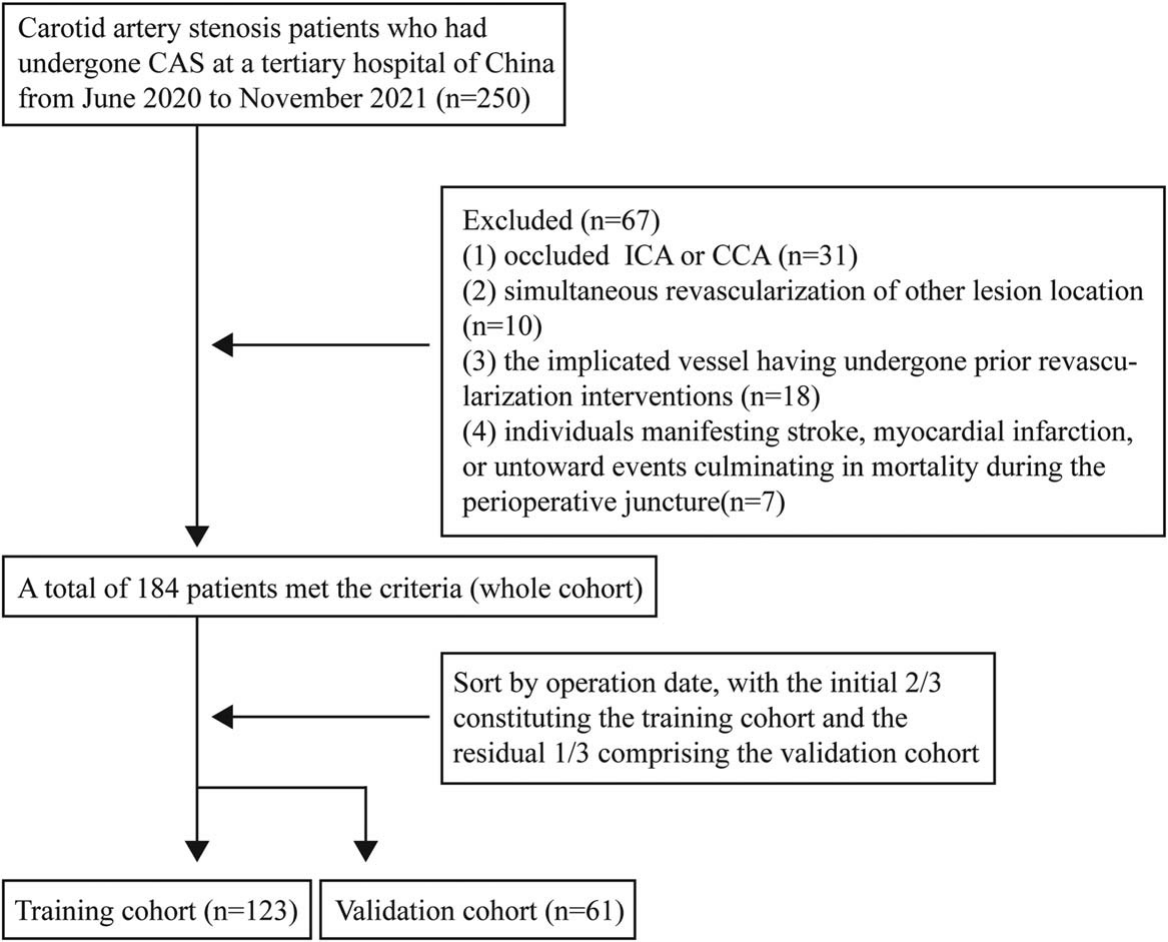
Study flow diagram.

The schematic representation of the study’s progression is depicted in Figure 1. A total of 250 CAS patients underwent preliminary screening, with 184 fulfilling the inclusion prerequisites. The training cohort encompassed 123 patients [median age, 64.22 (57.06–68.39) years; 90.24% male], while the validation cohort comprised 61 patients [median age, 64.85 (58.37–68.98) years; 88.52% male]. Furthermore, no statistical variances were discerned between the two cohorts concerning foundational demographics (Table 1). Within the training cohort, the SCI and normal groups consisted of 38 (30.89%) and 85 (69.11%) participants, respectively. The foundational demographics contrasting the SCI and normal groups are elucidated in Table 2. Individuals within the SCI group exhibited an advanced age [67.21 (64.58–75.96) vs. 60.48 (56.08–67.08), P<0.001], a heightened female composition (6, 15.79 vs. 6, 7.06%; *P*=0.186), a higher rate of asymptomatic lesion (28, 73.68 vs. 41, 48.24%, *P*=0.011) and an elevated BMI [26.57 (24.62–28.74) vs. 24.98 (23.56–27.16); *P*=0.022]. Additionally, no pronounced disparities were detected between the two cohorts concerning hypertension (30, 78.95% vs. 59, 69.41%; *P*=0.383), diabetes (12, 31.58% vs. 26, 30.59%; P>0.999), hyperlipidemia (20, 52.63% vs. 29, 34.12%; *P*=0.073), coronary artery disease (8, 21.05% vs. 12, 14.11%; *P*=0.280), atrial fibrillation (1, 2.63% vs. 1, 1.18%; *P*=0.524), bronchial asthma (1, 2.63% vs. 1, 1.18%; *P*=0.524), the % of stenosis of qualifying artery [82.07 (76.98–88.60) vs. 83.72 (77.21–90.00); *P*=0.493], or the length of the stenotic segment [22.15 (18.30–26.43) vs. 23.80 (19.55–28.20); *P*=0.311]. Neither tobacco consumption (*P*=0.294) nor alcohol intake (*P*=0.419) were deemed determinants for SCI.

**Table 1:** Demographic and anatomical characteristics of training and validation cohorts

Baseline characteristics	Training cohort (*N*=123)	Validation cohort (*N*=61)	*P*
Demographics			
Age, years	64.22 (57.06–68.39)	64.85 (58.37–68.98)	0.695
Sex			
Male	111 (90.24)	54 (88.52)	0.798
Female	12 (9.76)	7 (11.48)	
Symptomatic lesion	69 (56.10)	40 (65.57)	0.265
BMI	25.71 (23.78–27.56)	25.01 (22.95–26.97)	0.252
Hypertension	89 (72.36)	47 (77.05)	0.594
Diabetes	38 (30.89)	19 (31.15)	>0.999
Hyperlipidemia	49 (39.84)	24 (39.34)	>0.999
Coronary artery disease	20 (16.26)	7 (11.48)	0.508
Atrial fibrillation	2 (1.63)	1 (1.64)	>0.999
Bronchial asthma	2 (1.63)	1 (1.64)	>0.999
Smoking			
Never	50 (40.65)	30 (49.18)	
Former	28 (22.76)	13 (21.31)	0.518
Current	45 (36.59)	18 (29.51)	
Drinking			
Never	74 (60.16)	32 (52.46)	
Former	15 (12.20)	3 (4.92)	0.066
Current	34 (27.64)	26 (42.62)	
Aortic arch characteristics			
Aortic arch type			
I	68 (55.28)	33 (54.10)	
II	29 (23.58)	12 (19.67)	0.686
III	26 (21.14)	16 (26.23)	
Aortic arch variants	13 (10.57)	8 (13.11)	0.511
Aortic arch calcification			
None	50 (40.65)	20 (32.79)	
Mild	39 (31.71)	23 (37.70)	0.629
Moderate	26 (21.14)	12 (19.67)	
Severe	8 (6.50)	6 (9.84)	
Carotid artery characteristics			
Side of the artery, left	57 (46.34)	32 (52.46)	0.531
CCA ostium lesions	27 (21.95)	10 (16.39)	0.376
Diameter of CCA above clavicle, mm	7.35 (6.54–8.00)	7.13 (6.55–8.10)	0.877
Depth of CCA above clavicle, cm	3.40 (2.96–3.78)	3.44 (3.02–3.77)	0.858
Length of CCA from clavicle to CB, cm	7.80 (7.06–8.68)	7.82 (7.00–8.70)	0.898
ICA diameter at target lesion, mm	5.33 (4.71–6.26)	5.12 (4.58–5.80)	0.402
ICA diameter at skull base, mm	4.39 (3.76–4.95)	4.27 (3.70–4.90)	0.815
Proximal TI	65.31±121.20	65.31±109.20	0.227
Distal TI	134.70 (99.40–178.80)	119.60 (88.25–169.00)	0.252
Tandem CCA or MCA stenosis	35 (28.46)	14 (22.95)	0.427
Stenosis of qualifying artery (%)	83.00 (77.00–90.00)	83.33 (77.00–88.37)	0.658
Length of the stenotic segment, mm	23.10 (19.20–27.60)	24.50 (19.60–29.85)	0.384

**Table 2:** Baseline characteristics between SCI and normal group of training cohort

Baseline characteristics	SCI group (*N*=38)	Normal group (*N*=85)	*P*
Demographics			
Age, years	67.21 (64.58–75.96)	60.48 (56.08–67.08)	<0.001
Sex			
Male	32 (84.21)	79 (92.94)	0.186
Female	6 (15.79)	6 (7.06)	
Symptomatic lesion	28 (73.68)	41 (48.24)	0.011
BMI	26.57 (24.62–28.74)	24.98 (23.56–27.16)	0.022
Hypertension	30 (78.95)	59 (69.41)	0.383
Diabetes	12 (31.58)	26 (30.59)	>0.999
Hyperlipidemia	20 (52.63)	29 (34.12)	0.073
Coronary artery disease	8 (21.05)	12 (14.11)	0.280
Atrial fibrillation	1 (2.63)	1 (1.18)	0.524
Bronchial asthma	1 (2.63)	1 (1.18)	0.524
Smoking			
Never	14 (36.84)	36 (42.35)	
Former	12 (31.58)	33 (38.82)	0.294
Current	12 (31.58)	16 (18.82)	
Drinking			
Never	22 (57.89)	52 (61.18)	
Former	3 (7.89)	12 (14.12)	0.419
Current	13 (34.21)	21 (27.71)	
Aortic arch characteristics			
Aortic arch type			
I	14 (36.84)	54 (63.53)	
II	10 (26.32)	19 (22.35)	0.007
III	14 (36.84)	12 (14.12)	
Aortic arch variants	10 (26.32)	3 (3.53)	<0.001
Aortic arch calcification			
None	9 (23.68)	41 (48.24)	
Mild	10 (26.32)	29 (34.12)	0.002
Moderate	14 (36.84)	12 (14.12)	
Severe	5 (13.16)	3 (3.53)	
Carotid artery characteristics			
Side of the artery, left	19 (50.00)	38 (44.71)	0.696
CCA ostium lesions	17 (44.74)	10 (11.76)	<0.001
Diameter of CCA above clavicle, mm	7.71 (6.97–8.40)	7.19 (6.47–7.84)	0.024
Depth of CCA above clavicle, cm	3.34 (2.55–3.77)	3.42 (2.98–3.84)	0.262
Length of CCA from clavicle to CB, cm	7.71 (7.04–8.57)	7.80 (7.04–8.77)	0.474
ICA diameter at target lesion, mm	5.16 (4.55–6.29)	5.44 (4.75–6.18)	0.867
ICA diameter at skull base, mm	4.74 (4.01–5.08)	4.22 (3.66–4.90)	0.061
Proximal TI	115.60 (83.20–213.60)	102.70 (69.50–147.70)	0.014
Distal TI	166.90 (122.70–203.40)	118.50 (88.25–162.70)	<0.001
Tandem CCA or MCA stenosis	13 (34.21)	22 (25.88)	0.390
Stenosis of qualifying artery (%)	82.07 (76.98–88.60)	83.72 (77.21–90.00)	0.493
Length of the stenotic segment, mm	22.15 (18.30–26.43)	23.80 (19.55–28.20)	0.311

Pertaining to aortic arch attributes, disparities in aortic arch type (*P*=0.007), aortic arch variations (*P*<0.001), and aortic arch calcification (*P*=0.002) were statistically notable between the cohorts (Table 2). Relative to carotid artery attributes, a salient variance in the CCA ostium lesions (*P*<0.001) was discerned between cohorts. The median values delineating the diameter of the CCA above clavicle [7.71 (6.97–8.40) vs. 7.19 (6.47–7.84); *P*=0.024], proximal TI [115.60 (83.20–23.60) vs. 102.70 (69.50–147.70); *P*=0.014], and distal TI [166.90 (122.70–203.40) vs. 118.50 (88.25–162.70); *P*<0.001] were notably elevated within the SCI group in comparison to the normal group. Concurrently, the arterial side (left, 19, 50.00% vs. 38, 44.71%), the depth of the CCA above the clavicle [3.34 (2.55–3.77) vs. 3.42 (2.98–3.84); *P*=0.262], length of CCA from clavicle to carotid bifurcation [7.71 (7.04–8.57) vs.7.80 (7.04–8.77); *P*=0.474], ICA diameter at target lesion [5.16 (4.55–6.29) vs.5.44 (4.75–6.18); *P*=0.867], ICA diameter at skull base [4.74 (4.01–5.08) vs. 4.22 (3.66–4.90); *P*=0.061] and the occurrence of tandem CCA or MCA stenosis (13, 34.21% vs. 22, 25.88%; *P*=0.390) did not manifest significant variances between the two cohorts (Table 2). Then, by analyzing stent use, stent type (*P*=0.821) and size (*P*=0.681) were not considered confounders for SCI post-CAS (Supplementary Table S1, Supplemental Digital Content 2, http://links.lww.com/JS9/C804). In addition, we analyzed the mechanisms of SCI post-CAS. Outcomes manifested that postoperative ischemic stroke was predominantly ipsilateral, with artery-to-artery embolism being the chief mechanistic factor (Supplementary Table S2, Supplemental Digital Content 3, http://links.lww.com/JS9/C805). Finally, we categorized the variations into three groups. A: the common origin of the left common carotid artery and the brachiocephalic trunk or origin of the carotid artery from the brachiocephalic trunk; B: the left vertebral artery can arise from the aortic arch; and C: other variations. The proportion of A variant (85.71%) was greater than that of B (14.29%) and C variant (0.00%) (Supplementary Table S3, Supplemental Digital Content 4, http://links.lww.com/JS9/C806).

**Figure 2:**
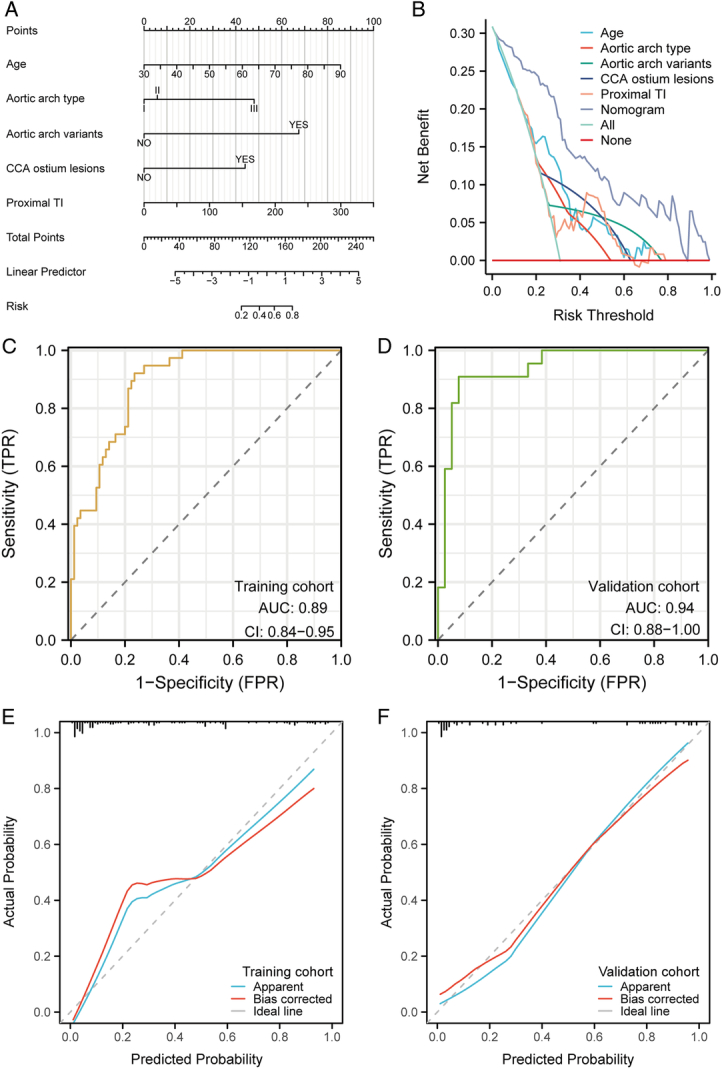
The nomogram for predicting the likelihood of SCI post-CAS and its validation and assessment. (A) Nomogram derived from the results of multivariable logistic analysis of the training cohort. (B) Decision curve analysis for the nomogram within the training cohort. (C-D) The outcomes of the ROC trajectory of the nomogram in both the training and validation cohorts. (E-F) Calibration graph of the nomogram in both the training and validation cohorts.

**Figure 3:**
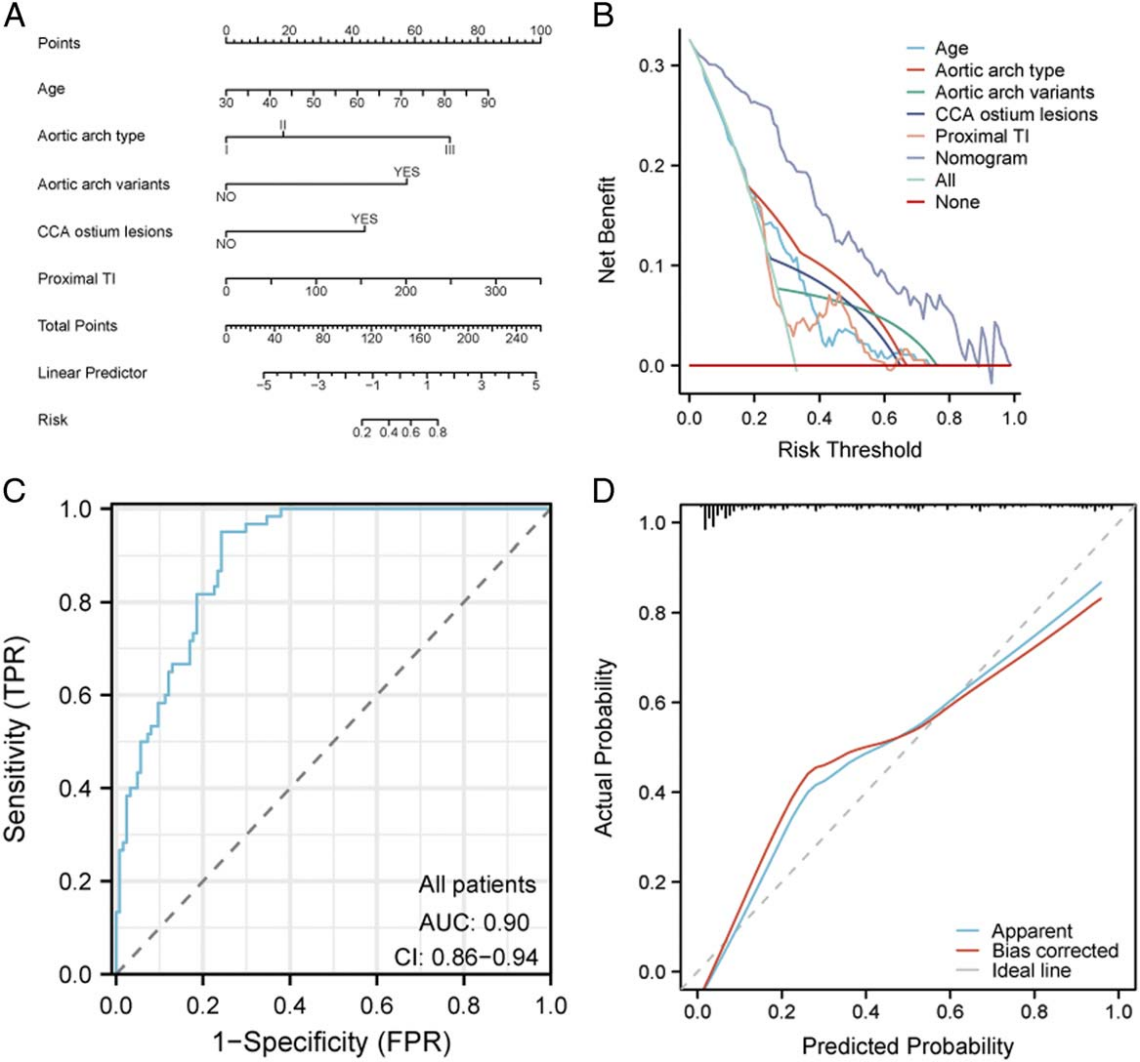
The nomogram of the composite cohort and its evaluation. (A) Nomogram pertaining to the composite cohort. (B) Decision curve analysis for the nomogram within the composite cohort. (C) The outcomes of the ROC trajectory for the nomogram in the composite cohort. (D) Calibration graph of the nomogram within the composite cohort.

The outcomes from the logistic regression analysis within the training cohort are presented in Table 3. Age (OR 1.08, 95% CI: 1.01–1.16; *P*=0.034), aortic arch type (III vs. I: OR 10.79, 95% CI: 2.12–54.81; *P*=0.005), aortic arch variants (OR 47.71, 95% CI: 6.05–376.09; *P*<0.001), CCA ostium lesions (OR 6.93, 95% CI: 1.49–32.32; *P*=0.014), and proximal TI (OR 1.01, 95% CI: 1.00–1.02; *P*=0.029) were discerned as independent determinants for SCI post-CAS. In the multivariate logistic regressions, type I aortic arch was used as the reference. No salient disparity was observed between types II and I, whereas a marked statistical variance was perceived between types III and I. Consequently, type III exhibited a heightened propensity towards SCI compared to types I and II. Predicated upon the five anatomical risk determinants, the nomogram was formulated with a C-index of 0.89 (95% CI: 0.84–0.95) (Fig. 2A). The DCA curve illustrated that the nomogram yielded a superior net advantage when juxtaposed with both the ‘all’ and ‘none’ paradigms and surpassed each predictor in terms of net benefit (Fig. 2B). The ROC curves of both the training cohort (AUC=0.89) and validation cohort (AUC=0.94) testify to the commendable risk prediction efficacy of the nomogram (Fig. 2C-D). The calibration curves for both cohorts denote the admirable discriminative prowess and calibration of the nomogram. The azure trajectory delineates the nomogram’s performance, whilst the crimson trajectory provides a correction for potential biases. In juxtaposition, the dashed trajectory symbolizes the ideal benchmark for a flawless nomogram (Fig. 2E-F). Additionally, within the entire cohort, the outcomes of the foundational data (Table 4) and logistic regression analysis (Table 5) align seamlessly with the training cohort. Furthermore, the comprehensive cohort also corroborates that the elaboration of a nomogram based on the aforementioned five autonomous determinants exhibits sterling predictive capacity (Fig. 3).

**Table 3:** Logistic regression analysis results of training cohort

Characteristics	OR (95% CI) univariate analysis	*P* value univariate analysis	OR (95% CI) multivariate analysis	*P* value Multivariate analysis
Age	1.09 (1.04–1.15)	<0.001	1.08 (1.01–1.16)	0.034
Manifestation				
Asymptomatic	Reference		Reference	
Symptomatic	3.01 (1.30–6.95)	0.011	1.24 (0.36–4.21)	0.736
Aortic arch type				
I	Reference		Reference	
III	4.50 (1.71–11.87)	0.002	10.79 (2.12–54.81)	0.005
II	2.03 (0.77–5.33)	0.151	1.08 (0.24–4.78)	0.919
Aortic arch variants				
No	Reference		Reference	
YES	9.76 (2.51–38.02)	0.001	47.71 (6.05–376.09)	< 0.001
Aortic arch calcification				
None	Reference		Reference	
Moderate	5.32 (1.85–15.28)	0.002	1.13 (0.20–6.42)	0.885
Severe	7.59 (1.53–37.72)	0.013	0.85 (0.05–13.86)	0.906
Mild	1.57 (0.57–4.35)	0.385	0.52 (0.10–2.26)	0.443
CCA ostium lesions				
No	Reference		Reference	
YES	6.07 (2.42–15.22)	<0.001	6.93 (1.49–32.32)	0.014
BMI	1.16 (1.02–1.33)	0.028	1.17 (0.93–1.47)	0.182
Diameter of CCA above clavicle, mm	1.60 (1.09–2.35)	0.017	1.22 (0.66–2.26)	0.534
Proximal TI	1.01 (1.00–1.02)	0.002	1.01 (1.00–1.02)	0.029
Distal TI	1.01 (1.01–1.02)	0.001	1.01 (1.00–1.02)	0.153

**Table 4:** Baseline characteristics between SCI group and normal group of the whole cohort

Baseline characteristics	SCI group (*N*=60)	Normal group (*N*=124)	*P*
Demographics			
Age, years	67.18 (63.54–73.16)	62.35 (56.58–67.20)	<0.001
Sex			
Male	52 (86.7)	113 (91.1)	0.439
Female	8 (13.3)	11 (8.9)	
Symptomatic lesion	44 (73.33)	65 (51.18)	0.007
BMI	26.06 (23.63–27.97)	24.95 (23.43–27.10)	0.106
Hypertension	47 (78.3)	89 (71.8)	0.376
Diabetes	19 (31.7)	38 (30.6)	>0.999
Hyperlipidemia	27 (45.0)	46 (37.1)	0.337
Coronary artery disease	13 (21.7)	14 (11.3)	0.076
Atrial fibrillation	2 (3.3)	1 (0.8)	0.248
Bronchial asthma	1 (1.7)	2 (1.6)	>0.999
Smoking			
Never	24 (40.0)	56 (45.2)	
Former	17 (28.3)	24 (19.4)	0.390
Current	19 (31.7)	44 (35.5)	
Drinking			
Never	32 (53.3)	74 (59.7)	
Former	4 (6.7)	14 (11.3)	0.265
Current	24 (40.0)	36 (29.0)	
Aortic arch characteristics			
Aortic arch type			
I	18 (30.0)	83 (66.9)	
II	14 (23.3)	27 (21.8)	<0.001
III	28 (46.7)	14 (11.3)	
Aortic arch variants	16 (26.7)	5 (4.0)	<0.001
Aortic arch calcification			
None	13 (21.7)	57 (46.0)	
Mild	17 (28.3)	45 (36.3)	
Moderate	22 (36.7)	16 (12.9)	<0.001
Severe	8 (13.3)	6 (4.8)	
CCA ostium lesions	24 (40.0)	13 (10.5)	<0.001
Carotid artery characteristics			
Side of the artery, left	30 (50.0)	59 (47.6)	0.758
Diameter of CCA above clavicle, mm	7.72 (6.97–8.55)	7.08 (6.47–7.82)	<0.001
Depth of CCA above clavicle, cm	3.36 (2.98–3.77)	3.44 (2.96–3.79)	0.535
Length of CCA from clavicle to CB, cm	7.62 (6.97–8.55)	7.86 (7.06–8.72)	0.262
ICA diameter at target lesion, mm	5.14 (4.51–5.95)	5.29 (4.77–6.13)	0.366
ICA diameter at skull base, mm	4.57 (3.92–5.07)	4.24 (3.66–4.88)	0.049
Proximal TI	114.50 (73.93-198.20)	101.60 (67.80–144.00)	0.020
Distal TI	153.30 (118.70–200.60)	115.30 (87.45-162.60)	<0.001
Tandem CCA or MCA stenosis	21 (35.0)	28 (22.6)	0.079
Stenosis of qualifying artery (%)	81.17 (77.00–87.44)	84.65 (77.47–90.00)	0.070
Length of the stenotic segment, mm	22.80±5.53	24.06±5.59	0.151

**Table 5:** Logistic regression analysis results of the whole cohort

Characteristics	OR (95% CI) univariate analysis	*P* value univariate analysis	OR (95% CI) multivariate analysis	*P* value multivariate analysis
Age	1.08 (1.04–1.12)	<0.001	1.06 (1.00–1.12)	0.047
Aortic arch type				
I	Reference		Reference	
III	9.22 (4.07–20.93)	<0.001	21.14 (5.98–74.67)	<0.001
II	2.39 (1.05–5.44)	0.038	2.19 (0.70–6.80)	0.177
Aortic arch variants				
No	Reference		Reference	
YES	8.66 (2.99–25.03)	<0.001	15.04 (3.43–65.96)	<0.001
Aortic arch calcification				
None	Reference		Reference	
Moderate	1.66 (0.73–3.77)	0.228	0.73 (0.22–2.41)	0.608
Mild	6.03 (2.50–14.56)	<0.001	0.75 (0.18–3.12)	0.696
Severe	5.85 (1.73–19.76)	0.004	0.77 (0.10–5.88)	0.802
CCA ostium lesions				
No	Reference		Reference	
YES	5.69 (2.63–12.33)	<0.001	6.88 (1.88–25.28)	0.004
Diameter of CCA above clavicle, mm	1.74 (1.26–2.40)	<0.001	1.52 (0.97–2.37)	0.066
Proximal TI	1.01 (1.00–1.01)	0.002	1.01 (1.00–1.02)	0.028
Distal TI	1.01 (1.01–1.02)	<0.001	1.01 (1.00–1.01)	0.112

## Discussion

A great number of SCI occurs subsequent to CAS, which is purportedly associated with cognitive decline, dementia, and an augmented risk of ensuing stroke episodes^8–10,26^. Antecedent research has posited that specific vascular anatomical attributes act as determinants for SCI post-CAS^14^. Such anatomical characteristics serve as pivotal referential indices for surgeons undertaking CAS operations. Consequently, it is of paramount importance to devise an individualized evaluation paradigm predicated on anatomical attributes to inform CAS clinical practice. Within the purview of this study, we have proffered a reliable nomogram, predicated on age, aortic arch type, aortic arch variations, CCA ostium abnormalities, and proximal TI, to forecast the likelihood of SCI post-CAS. Through this nomogram, healthcare practitioners can institute more meticulous risk stratification tactics and tailored interventions for patients undergoing CAS to curtail the prevalence of postoperative SCI.

Many excellent studies have explored the causes of SCI post CAS. Some studies have identified risk factors by comparing interventional therapy with different vessels. Compared with CAS, vertebrobasilar stenting had a longer operation time and more residual stenosis, which also tends to correspond to more SCI^27^. Furthermore, long-term follow-up showed that in-stent restenosis was associated with regional infarction in CAS^28^. In the study of Muller MD *et al*.^29^, 51% (41/97) of patients had SCI post-CAS. This study suggested that the complex morphology of the aortic arch and the distortion of the internal carotid artery are risk factors for SCI after CAS. In addition, a systematic review showed that higher age, plaque susceptibility, and complex carotid/aortic arch anatomy are considered risk factors of SCI-post CAS^30^. These clinical predictors can help identify high-risk patients who may need additional preventive measures or alternative treatment strategies.

In this investigation, age, aortic arch type, aortic arch variants, CCA ostium lesions, and proximal TI were discerned to be independent determinants of SCI subsequent to CAS. Unfortunately, the underlying mechanism by which these risk factors lead to SCI post-CAS remains obscure. Initially, regarding age, prior studies have evinced that individuals of advanced age tend to exhibit heightened adverse event rates post-CAS in comparison to their younger counterparts. Moreover, geriatric patients might not derive tangible benefits from the employment of cerebral protective apparatuses^31^. As such, a more stringent perioperative oversight ought to be conferred upon the elderly demographic. Secondly, intricate or formidable anatomy might precipitate technical impediments, escalated contrast injection and fluoroscopy durations, tumultuous guidewire and catheter operations, and an extension in the aggregate procedure timeframe. The aforesaid elements are deemed pivotal antecedents to SCI post-CAS^15,16,32,33^. Concerning the aortic arch configuration, the augmented intricacy of the type III aortic arch, distinguished by an enhanced curvature and angularity vis-à-vis type I and II aortic arch, engenders complications in catheter operations and the traversal of the aortic arch^23,34^. It was reported that prolonged catheterization duration is associated with a higher risk for adverse outcomes during CAS treatment. However, the presence of arterial arch variants will bring a difficulty in placing a catheter into the target artery, extending the duration, and finally, the risk of perioperative complications increases^24^. Antecedent studies have illuminated that stent positioning in the CCA ostium complicates the circumvention of aortic protrusion. Furthermore, given the catheter’s positioning beneath the aortic arch, the guiding catheter might lose its stability during stent positioning^35^. These factors may lead to technical barriers, prolonged contrast injection and fluoroscopy times, disturbances in guidewire and catheter manipulation, and prolonged total procedure time. Terakado *et al*.^35^ advanced the proposition of a gooseneck snare balloon catheter lift methodology as an avenue to redress these issues, proffering an efficacious modus operandi for stent placement at the CCA ostium. Additionally, proximal TI has been demonstrated to be intrinsically linked with technical success and neurological complications of CAS, while distal TI remains innocuous to both outcomes^16^. The consonance between these findings and our study’s revelations reaffirms the rigor of our scholarly pursuit. An elevation in the proximal tortuosity index denotes an amplification in the contortion of the carotid artery, exacerbating the technical hurdles encountered during CAS procedures. This heightened complexity elevates the propensity for vessel wall impairment, culminating in deficient stent deployment and concomitant hemodynamic shifts.

The aforementioned intricate anatomical characteristics have been associated with an elevated risk of SCI post-CAS. Consequently, the formulation of an individualized comprehensive evaluation paradigm that contemplates these independent determinants becomes paramount for guiding practitioners in furnishing patients with more safeguarded therapeutic interventions. Within the training cohort, we devised a nomogram predicated upon the aforementioned five independent determinants for SCI post-CAS, boasting a C-index of 0.89 (95% CI: 0.84–0.95). Concurrently, both the validation cohort (C-index=0.94) and the composite cohort (C-index=0.90) manifested a commendable C-index. Generally speaking, a C-index >0.75 signifies robust discrimination, thereby attesting to the prowess of this nomogram^36^. DCA curves offer an assessment of the net advantage of a model by evaluating the differential between the quantum of true and false-positive outcomes and are ubiquitously employed in evaluating the potential enhancement of patient outcomes through nomogram-informed decisions^36^. The DCA trajectories elucidate that the nomogram predicts the likelihood of SCI post-CAS, proffering a superior net advantage over isolated risk factors^37^. As prognosticated by this nomogram, in instances with elevated risk trajectories for SCI post-CAS, it may be propitious to engage more seasoned operators or contemplate alternate modalities such as carotid endarterectomy or transcarotid revascularization. Such strategic deployments may further refine patient outcomes and curtail the incidence of inauspicious events^38,39^. To the best of our understanding, the present investigation stands as a pioneering endeavor in leveraging a nomogram predicated upon anatomical attributes to anticipate SCI post-CAS. This nomogram exhibits commendable performance across the training, validation, and composite cohorts, and furnishes invaluable perspectives conducive to clinical praxis and the identification of high-risk patients who may necessitate enhanced precautionary measures or alternate therapeutic blueprints.

This study innovatively demonstrated that age, aortic arch type, aortic arch variations, CCA ostium lesions, and proximal TI are independent determinants associated with CAS after SCI. Nomograms based on these risk factors performed exemplary in training, validation, and composite cohorts, have strong prognostic significance, and can be used as an important aid in clinical decision-making. However, it is imperative to acknowledge several inherent limitations. Primarily, this observational analysis is susceptible to endemic bias emanating from inescapable confounding variables. Secondly, our study’s retrospective evaluation of a singular-center database imparts constraints on external validity. Hence, a multi-institutional validation study becomes a sine qua non to ascertain the efficacy of the nomogram before its integration into clinical routines. Secondly, the outcomes were presented with a notably expansive 95% CI. While a broad CI might insinuate ambiguity, it should not be construed pejoratively. It is quintessential to articulate and interpret the CI with integrity, contemplating its ramifications vis-à-vis the study’s results. Prospectively, the accrual of additional data or the undertaking of supplementary analyses is imperative to contract the breadth of the CI, thereby augmenting the precision of the projections. Thirdly, some models, such as machine learning^40,41^, have potential and value in prognostic prediction of patients and can be utilized to further evaluate our study in the future. Lastly, our investigation, being retrospective in nature, did not encompass catheterization durations. The nexus between catheterization duration and multifarious anatomical attributes warrants scrupulous exploration in impending prospective research endeavors.

## Conclusions

To encapsulate, this investigation discerned age, aortic arch type, aortic arch variations, CCA ostium lesions, and proximal TI as autonomous risk determinants for SCI subsequent to CAS. Premised on these determinants, a propitious nomogram was conceived, which demonstrated exemplary performance across the training, validation, and whole cohorts. The formulated nomogram makes it possible for clinicians to quickly understand and apply the results of the model, and can be used as an important aid in clinical decision-making. In addition, subsequent research endeavors are mandated to externally authenticate the nomogram across diverse populations.

## Ethical approval

The study procedures were approved by Ethics Committee of Xuanwu Hospital, Capital Medical University. Number: [2021]124.

## Consent

Written informed consent was obtained from the patient for publication and any accompanying images. A copy of the written consent is available for review by the Editor-in-Chief of this journal on request.

## Source of funding

This study was supported by Research and translation application of clinical diagnosis and treatment technology of the capital (Z201100005520020; Z201100005520019) and Beijing Hospitals Authority’s Ascent Plan (DFL20220702).

## Author contribution

T.L. and R.Y.: conceptualization; T.L. and J.W.: data curation; T.L., R.Y., and J.W.: formal analysis; B.Y. and L.J.: funding acquisition; T.l., R.X., and A.D.: investigation; T.L., G.L., J.J., and X.B.: methodology; T.L.: project administration; T.W., T.L., and Y.W.: resources; T.L.: software; T.W., T.L., and Y.W.: supervision; T.L.: validation; T.L. and T.L.: visualization; T.L., B.Y., and L.J.: writing – original draft; T.L., R.Y., J.W., T.W., A.D., B.Y., and L.J.: writing – review and editing.

## Conflicts of interest disclosure

The authors declare no conflicts of interest.

## Research registration unique identifying number (UIN)

1. Name of the registry: A new evaluation system for carotid atherosclerotic stenosis based on molecular imaging and computational fluid dynamics technology.
2. Unique identifying number or registration ID: ChiCTR2100051697.
3. Hyperlink to your specific registration (must be publicly accessible and will be checked): https://www.chictr.org.cn/index.html.

## Guarantor

Liqun Jiao MD. Department of Neurosurgery, Xuanwu Hospital, Capital Medical University, No.45 Changchun Street, Xicheng District, Beijing 100053, China. E-mail: liqunjiao@sina.cn.

## Data availability statement

The data that support the study findings are available upon reasonable request from the corresponding authors.

## Provenance and peer review

Not commissioned, externally peer-reviewed.

## Supplementary Material

SUPPLEMENTARY MATERIAL
