# Supplementary material for: Anatomical predispositions for silent cerebral infarction postcarotid artery stenting: a retrospective cohort
Source: Int J Surg. 2024 Jun 19;110(12):7889–99. doi: 10.1097/JS9.0000000000001833 (PMC11634115; doi:10.1097/JS9.0000000000001833)
Supplement: SUPPLEMENTARY MATERIAL [file js9-110-7889-s002.docx]

Table S1. Details of stent use

| Categories | SCI group（N=60） | Normal group（N=124） | P-value |
| --- | --- | --- | --- |
| Brand of stent | | |  |
| Wallstent | 34 (56.66) | 70 (56.45) |  |
| Precise | 12 (20.00) | 29 (23.39) | 0.821 |
| Acculink | 13 (21.67) | 23 (18.55) |  |
| Protégé | 1 (1.67) | 2 (1.61) |  |
| Diameter of the stent (mm) | | |  |
| 7 | 10 (16.67) | 13 (10.48) |  |
| 8 | 10 (16.67) | 23 (18.55) | 0.681 |
| 9 | 34 (56.66) | 73 (58.87) |  |
| 10 | 6 (10) | 15 (12.10) |  |
