# Supplementary material for: Anatomical predispositions for silent cerebral infarction postcarotid artery stenting: a retrospective cohort
Source: Int J Surg. 2024 Jun 19;110(12):7889–99. doi: 10.1097/JS9.0000000000001833 (PMC11634115; doi:10.1097/JS9.0000000000001833)
Supplement: SUPPLEMENTARY MATERIAL [file js9-110-7889-s003.docx]

Table S2. Mechanism of SCI post-CAS

| Mechanism | Number (N=60) |
| --- | --- |
| Perforator occlusive | 0 (0.00) |
| Artery-to-artery embolism | 30 (50.00) |
| Hypoperfusion | 11 (18.33) |
| Mixed | 19 (31.67) |
