# Supplementary material for: Anatomical predispositions for silent cerebral infarction postcarotid artery stenting: a retrospective cohort
Source: Int J Surg. 2024 Jun 19;110(12):7889–99. doi: 10.1097/JS9.0000000000001833 (PMC11634115; doi:10.1097/JS9.0000000000001833)
Supplement: SUPPLEMENTARY MATERIAL [file js9-110-7889-s004.docx]

Table S3. Proportion of different aortic arch variants

| Aortic arch variants | Number (N=21) |
| --- | --- |
| A | 18 (85.71) |
| B | 3 (14.29) |
| C | 0 (0.00) |

A: common origin of the left common carotid artery and the brachiocephalic trunk or origin of the carotid artery from the brachiocephalic trunk; B: The left vertebral artery can arise from the aortic arch; C: Other variations
